# A comparative analysis of the efficacy and safety of paricalcitol versus other vitamin D receptor activators in patients undergoing hemodialysis: A systematic review and meta-analysis of 15 randomized controlled trials

**DOI:** 10.1371/journal.pone.0233705

**Published:** 2020-05-29

**Authors:** Xinghua Geng, Ermin Shi, Shiwei Wang, Yuzhi Song

**Affiliations:** 1 Hemodialysis Room, The Fourth Central Hospital of Baoding City, Baoding, Hebei, China; 2 Administration Department, The Fourth Central Hospital of Baoding City, Baoding, China; University of Liège, BELGIUM

## Abstract

Paricalcitol, a new vitamin D receptor activator (VDRA), is reported to be more effective than other VDRAs in reducing calcium and phosphorus levels in patients undergoing hemodialysis. However, the efficacy and safety of paricalcitol remain controversial. This analysis compares paricalcitol with other VDRAs in patients undergoing hemodialysis. We searched the Cochrane Library, PubMed, EMBASE, Web of Science, and CNKI up to April 22, 2019. Standardized mean difference (SMD), risk ratio (RR) and 95% confidence interval (CI) values were estimated to compare the outcomes of the groups. Two reviewers extracted data and assessed trial quality independently. All statistical analyses were performed using the standard statistical procedures of RevMan 5.2 and Stata 12.0. Fifteen studies (*N* = 110,544) were included in this meta-analysis. Of these studies, 11 were randomized controlled trials (RCTs) and 4 were non-randomized studies of interventions (NRSIs). Patients receiving paricalcitol experienced better overall survival (OS) than patients receiving other VDRAs, with a pooled hazard ratio of 0.86 (95% CI 0.80–0.91; *P* < 0.00001). Intact parathyroid hormone (iPTH) levels were significantly reduced in the paricalcitol group compared to the group receiving other VDRAs, with a pooled SMD of -0.53 (95% CI -0.89– -0.16; *P* = 0.004). There was a significant increase in serum calcium levels from baseline in the paricalcitol group compared to the other VDRAs group when limiting the analysis to RCTs, with a pooled SMD of 2.14 (95% CI 0.90–3.38; *P* = 0.0007). Changes in serum calcium levels were significantly lower in the paricalcitol group when the analysis was limited to NRSIs, with a pooled SMD of -0.85 (95% CI -1.34–-0.35; *P* = 0.0008). The NSRI analysis also showed a significant reduction in serum phosphorus levels in the paricalcitol group, with a pooled SMD of -0.57 (95% CI -1.00–-0.13; *P* = 0.01). No significant differences were observed in the incidence of hypercalcemia, hyperphosphatemia, or adverse events. Generally, paricalcitol seems superior to other VDRAs in reducing mortality and iPTH levels in patients undergoing hemodialysis. However, the comparative effectiveness of paricalcitol in reducing serum calcium and phosphorus levels needs further exploration. No significant difference was found in the rate of adverse events.

## Introduction

Chronic kidney disease (CKD) is associated with numerous complications, including secondary hyperparathyroidism (SHPT), bone disorders, and cardiovascular disease [1–4]. These are the main causes of dialysis-related mortality [1–4]. Although therapeutic advances have been made in recent years, patients with stage 5 CKD maintained on hemodialysis still have a higher mortality rate [5]. Hemodialysis is a life-sustaining therapy for patients with end-stage renal disease (ESRD). It is currently used by close to 400,000 patients in the United States, who represent almost 90% of the ESRD population [6]. Chronic kidney disease patients have lower levels of 1α,25-dihydroxyvitamin D3, resulting in decreased intestinal calcium absorption, increased parathyroid hormone (PTH) production, and dysregulation of phosphorus metabolism [7–10].

Hyperparathyroidism occurs in most patients during the progression of CKD. One catalyst of hyperparathyroidism is a reduction in serum levels of 1,25-dihydroxyvitamin D [1,25(OH)(2)D], as a result of a decrease in renal 1alpha hydroxylase activity, which converts 25-hydroxyvitamin D into its activated form [7, 11]. The combination of persistently high PTH and low 1,25(OH)(2)D is associated with bone loss, cardiovascular disease, immune suppression and increased mortality in patients with end-stage kidney failure. Consequently, maintaining sufficient levels of vitamin D is crucial for CKD patients with SHPT.

The vitamin D endocrine system plays an essential role in calcium homeostasis and bone metabolism. Research during the past two decades has revealed a diverse range of biological actions of vitamin D, including induction of cell differentiation, inhibition of cell growth, immunomodulation, and control of other hormonal systems [9]. Vitamin D is a prohormone that is metabolically converted into the active metabolite 1,25(OH)(2)D [12, 13]. This vitamin D hormone activates a cellular receptor (the vitamin D receptor, or VDR), which alters the transcription rates of the target genes responsible for biological responses [14].

Recent studies in dialysis patients suggest that paricalcitol, a selective vitamin D receptor activator (VDRA), is associated with lower morbidity, a higher survival rate, improved efficacy and a more favorable side-effect profile than calcitriol [15–29]. These findings have led to the hypothesis that systemic activation of VDRs has direct effects on the cardiovascular system, thereby decreasing mortality in CKD [30–32]. Current guidelines for regulating serum calcium, phosphate, and PTH recommend specific interventions at various stages of CKD to prevent or postpone irreversible parathyroid disease and decrease cardiovascular morbidity and mortality. However, emerging data suggest that vitamin D therapy may prolong survival in this patient population by mechanisms that are independent of calcium, phosphate, and PTH.

Previous meta-analyses mainly compared VDRAs (including paricalcitol) to placebo [33–35] in dialysis patients [35] and patients not requiring dialysis [33, 34, 36]. Both paricalcitol and other VDRAs have demonstrated benefits for patients undergoing hemodialysis. However, the safety and effectiveness of paricalcitol compared to other VDRAs have not been well studied. The present systematic review and meta-analysis was designed to compare paricalcitol to other VDRAs in a large sample of patients undergoing hemodialysis (*N* = 110,544).

## Methods and materials

### Criteria for considering studies

We included studies if they met the following criteria: a. randomized controlled trial (RCT) or observational study (prospective or retrospective); b. study population consisting of adult patients with CKD (5D) and SPHT who were undergoing hemodialysis; c. study intervention was paricalcitol therapy; d. comparator was a vitamin D analogue, such as calcitriol, alfacalcidol, or maxacalcitol; e. study outcomes included one or more of the following: percentage of participants with target reduction in intact parathyroid hormone (iPTH) from baseline; incidence of hypercalcemia, hyperphosphatemia, or elevated calcium phosphorus product; all-cause mortality; and end-of-treatment serum phosphorus, calcium, and iPTH levels.

Studies were excluded if they met the following criteria: a. experimental trial on animals or a non-human study; b. study population including patients with other endocrine diseases that would affect outcomes; c. study was an abstract, letter, editorial, expert opinion, review, or case report; or d. lack of sufficient data or failure to meet the inclusion criteria.

### Search strategy

We searched the Cochrane Library, PubMed, EMBASE, Web of Science, and CNKI databases to April 22, 2019. Our strategy was based on combinations of medical subject headings (MeSH) and the keywords were “hemodialysis”, “dialysis”, “paricalcitol”, “kidney failure”, “renal insufficiency”, “vitamin D” and “chronic kidney disease”. The detailed search strategy was showed in the appendix file. Two assessors independently screened the titles and abstracts of each study. Their disagreement was solved by discussion. When a relevant study was identified, its full text was obtained for further evaluation. The full text of related references was also obtained for review. References that met the inclusion criteria were also included in the meta-analysis.

### Quality assessment and data extraction

Two reviewers assessed the quality of each RCT using the previously validated 5-point Jadad scale [37]. The disagreement was solved by their discussion. Studies with scores of 3 or more were considered high quality. The 9-star Newcastle-Ottawa Scale (NOS) was used to assess the quality of non-randomized studies of interventions (NRSIs) [38]. Studies with scores ≥ 6 were considered high quality. In addition, the risk of bias [39], for each individual study and across all studies, was evaluated and graphically displayed in figures generated by RevMan 5.2 software [40].

Data for the comparative analysis of paricalcitol versus other VDRAs, for patients undergoing hemodialysis, were extracted independently by two reviewers. Disagreements were resolved through discussion. The extracted data included first author, year of publication, sample size, intervention, age, follow-up time, and study quality score and outcomes. These data were standardized and input into RevMan 5.2 software for analysis [40].

### Statistical analysis

Data on study outcomes in the paricalcitol group and the other VDRAs group were combined and analyzed using the standard statistical procedures of RevMan 5.2 [40]. Dichotomous outcomes were compared based on the risk ratio (RR) and continuous outcomes were compared in terms of the standardized mean difference (SMD). The *P*_*h*_ value and *I*^*2*^ statistic (ranging from 0% to 100%) derived the chi-square-based Q test [41] were used to assess the heterogeneity between studies. A *P*_*h*_ ≤ 0.10 was deemed to represent significant heterogeneity [42]; in such cases, pooled RRs were estimated using a random effects model (the DerSimonian and Laird method [43]). When heterogeneity was not observed (*P*_*h*_ > 0.10), a fixed effects model (the Mantel–Haenszel method [44]) was used. Differences in outcome measures were considered statistically significant if the 95% CI of the pooled RR did not cross 1, or if the 95% confidence interval (CI) of the pooled SMDs did not cross 0.

Regarding the pooled SMD estimates of the reduction in iPTH from baseline, we performed subgroup analysis by route of administration (intravenous, oral), sample size (≤ 100 pts, > 100 pts), baseline iPTH level (≤ 68.4 pmol/L, > 68.4 pmol/L), and Jadad score (≤ 2, > 2). In addition, sensitivity analysis was performed to examine the stability of the overall results, and to identify sources of heterogeneity. Sensitivity analysis was conducted by removing each study from the analysis in turn to observe the effect of each single study on the pooled result. Finally, we checked for publication bias using Begg’s funnel plots and Egger’s publication bias plots. If the shape of a funnel plot was not obviously asymmetrical, we concluded that there was no obvious publication bias. All statistical analyses were performed using the standard statistical procedures of RevMan 5.2 and Stata 12.0.

This systematic review and meta-analysis followed the Preferred Reporting Items for Systematic Reviews and Meta-Analyses (PRISMA) guidelines [45] and has been assessed in line with AMSTAR (Assessing the methodological quality of systematic reviews) Guidelines [46].

## Results and discussion

### Characteristics and quality assessment of the included studies

**Fig 1:**
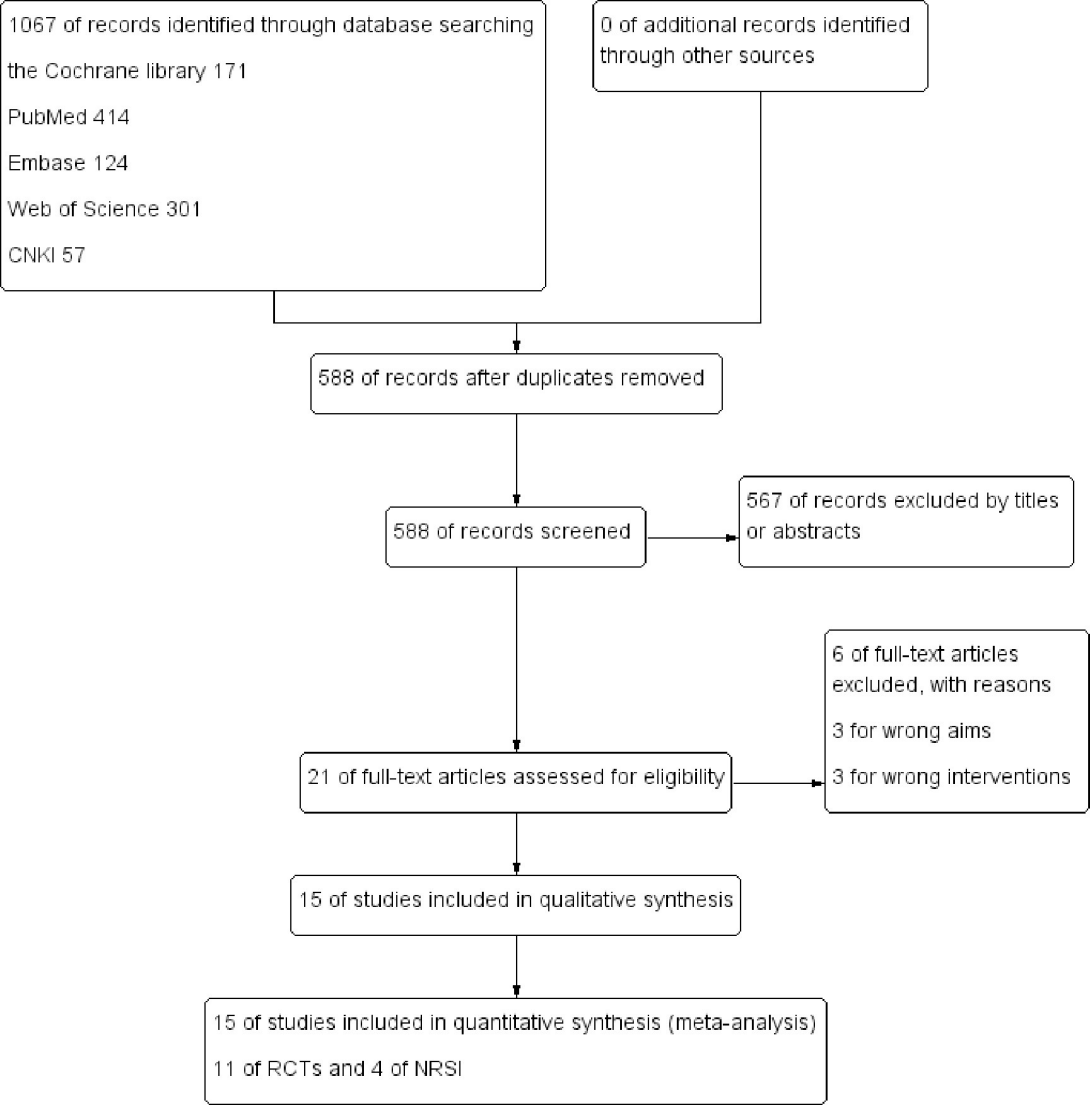
Flow diagram of literature search and selection of included studies for meta-analysis.

The initial search generated 1,067 records. After removal of duplicates, 588 records remained, of which 567 were excluded after screening the title and abstracts. Following full-text review of the 21 studies chosen for further evaluation, 6 studies [Ref. 1–6] were excluded further and 15 studies ([15, 16–29]; *N* = 110,544) that met the inclusion criteria were included in the final analysis. Of the 15 included studies, 11 were RCTs (*N* = 1,086) and 4 were NRSIs (*N* = 109,458). Details of the search process and a summary of the studies are shown in the study flow diagram (Fig 1). Other study characteristics are shown in Table 1.

**Table 1:** The characteristics of included studies for the present systematic review and meta-analysis.

Study (author / year)	Country	Sample size (pts) Paricalcitol/VDRA	Age (year) (mean±SD)	Interventions	Male / female	Follow-up time	Outcomes	Score
**RCTs**								**Jadad**
Abdul Gafor AH, et al.2009	Malaysia	13 / 12	48.2±14.1 / 47.8±16.4	Paricalcitol vs Calcitriol	14 / 11	12 weeks	Serum PTH, phosphorus, calcium	2
Akizawa T, et al.2015	USA	127 / 128	61.5±11.2 / 61.6±12.5	Paricalcitol vs Maxacalcitol	163 / 92	12 weeks	Reduction of iPTH, Hypercalcemia, Hyperphosphatemia	4
Farhat K, et al.2018	the Netherlands	14 / 13	61.7±10.2 / 62.3±15.4	Paricalcitol vs Calcitriol	23 / 4	24 weeks	Parameters, and safety	4
Hansen D, et al.2011	Denmark	42 / 38	63.7±15.8 / 63.7±14.0	Paricalcitol vs Alfacalcidol	51 / 29	16 weeks	Reduction of iPTH, Hypercalcemia, Hyperphosphatemia, Ca-P product, serum PTH, phosphorus, calcium	3
Jamaluddin EJ, et al.2014	Malaysia	12 / 14	48.33±12.05 / 39.07±12.67	Paricalcitol vs Calcitriol	13 / 13	15 weeks	Reduction of iPTH, Hypercalcemia	2
Ketteler M, et al.2012	USA	134 / 134	61.2±12.7 / 59.9±12.0	Paricalcitol vs Cinacalcet	168 / 100	28 weeks	Control of iPTH	4
Lund RJ, et al.2010	USA	9 / 9	51.1	Paricalcitol vs Calcitriol		5 weeks	Serum phosphorus, calcium	4
Ong LM, et al.2013	Malaysia	36 / 30	46.3±13.1 / 45.4±17.9	Paricalcitol vs Calcitriol	41 / 25	24 weeks	Reduction of iPTH, Hypercalcemia, Ca-P product, serum phosphorus, calcium	2
Sprague SM, et al.2001	USA	19 / 19	NR	Paricalcitol vs Calcitriol	NR	every 4 weeks	Serum PTH, calcium, and phosphorus	3
Sprague SM, et al.2003	USA	130 / 133	56.7	Paricalcitol vs Calcitriol	NR	32 weeks	Reduction of iPTH, Hypercalcemia, Hyperphosphatemia, Ca-P product	**4**
Veceric-HZ, et al.2016	Slovenia	10 / 10	56 / 50	Paricalcitol vs Calcitriol	15 / 5	12 weeks	PTH suppression, Ca and P level and calcium-phosphorus product	1
**NRSI**								**NOS**
Cozzolino M, et al.2012	UK	1,630 / 823	68	Paricalcitol vs Calcitriol	1531 / 922	over 2-year	Time-to-death, iPTH	7
Shinaberger CS, et al.2008	USA	23,727 / 10,580	60.8±14.8 / 61.8±15.6	Paricalcitol vs Calcitriol	18394 / 15913	3 years	The 3-yr mortality, serum iPTH	6
Teng M, et al.2003	USA	29,021 / 38,378	60.7 / 61.3	Paricalcitol vs Calcitriol	35431 / 31968	36 months	The mortality rate, calcium and phosphorus levels	7
Tentori F, et al.2006	USA	2,087 / 3,212	61 / 62	Paricalcitol vs Calcitriol / Doxercalciferol	3854 / 3877	37 weeks	Mortality rates	7

VDRA, vitamin D receptor activator; iPTH, intact parathyroid hormone; RCT, randomized controlled trial; NRSI, non-randomized studies of intervention; NOS, Newcastle-Ottawa Scale; NR, not report; Ca, calcium; P, phosphorus.

**Fig 2:**
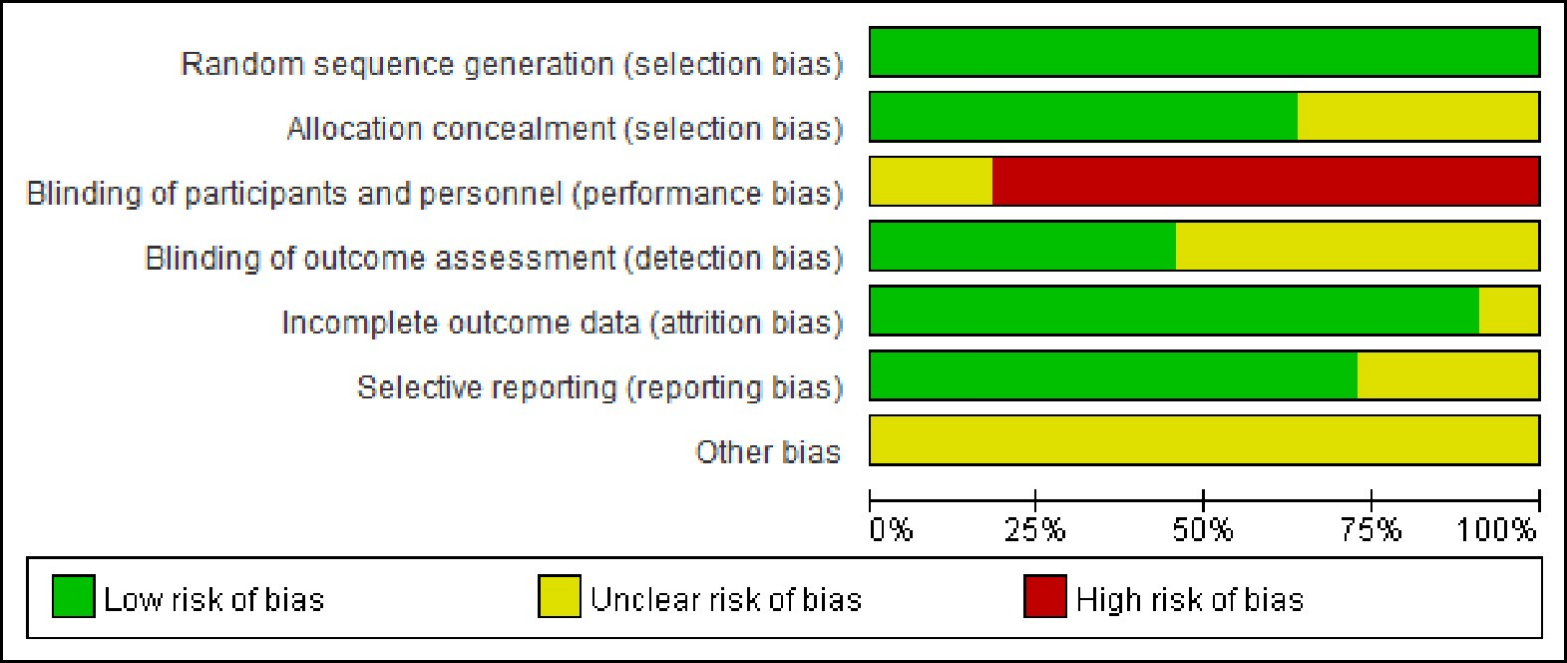
Risk of bias graph: Review authors' judgements about each risk of bias item presented as percentages across all included studies.

**Fig 3:**
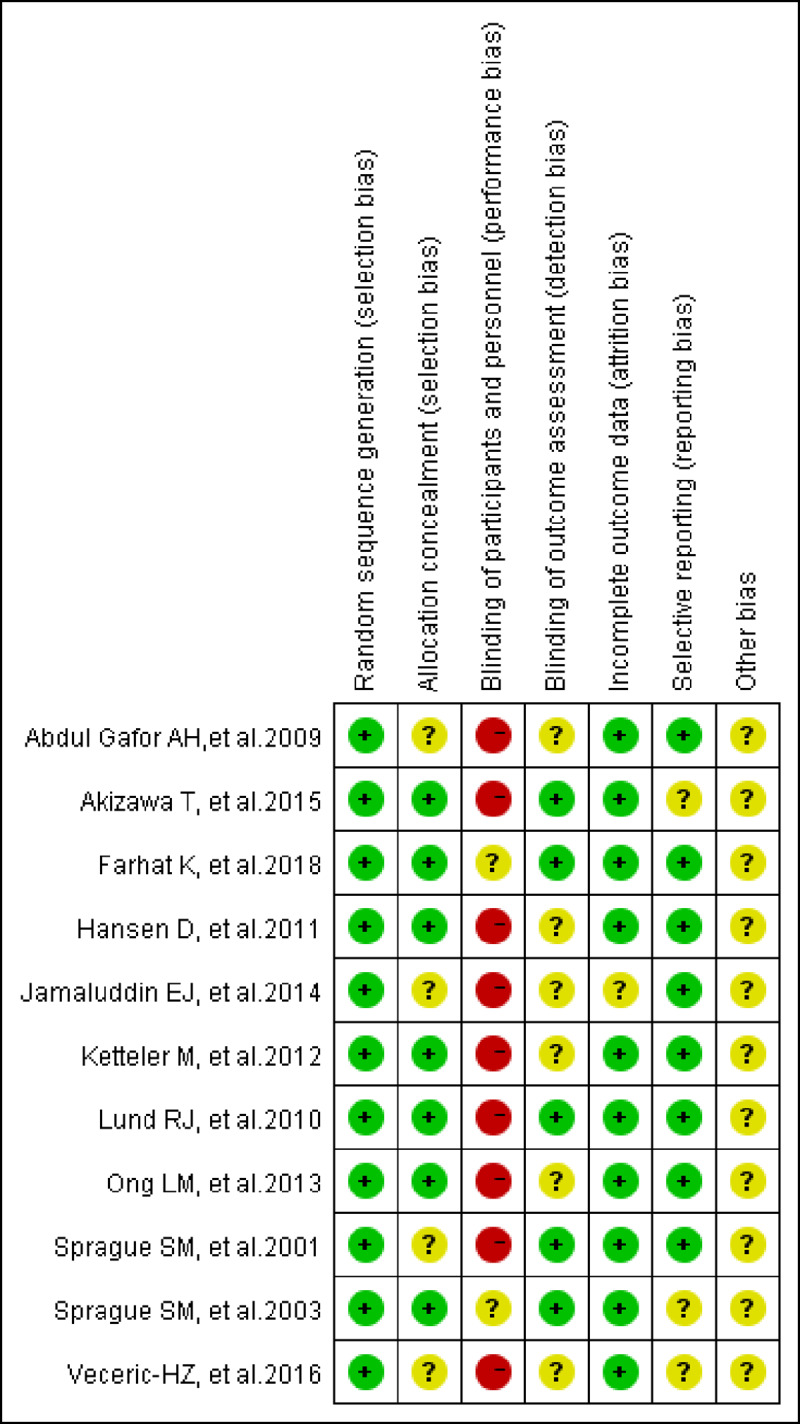
Risk of bias summary: Review authors' judgements about each risk of bias item for each included study.

Seven of eleven RCTs (63.6%) were given a Jaded score of ≥ 3 and classified as high quality. All NRSIs received a NOS score ≥ 6 and were classified as high quality. Risk-of-bias graphs were generated for the included studies. The data on risk of bias for each RCT, and across RCTs, are presented as percentages in Figs 2 and 3. The risk-of-bias graphs indicated generally good methodological quality. All RCTs showed low risk of bias on the item “Random sequence generation (selection bias)”. There was a high risk of performance bias and unclear risk of detection bias and other biases.

### Comparison of overall survival between paricalcitol and other VDRAs

**Fig 4:**
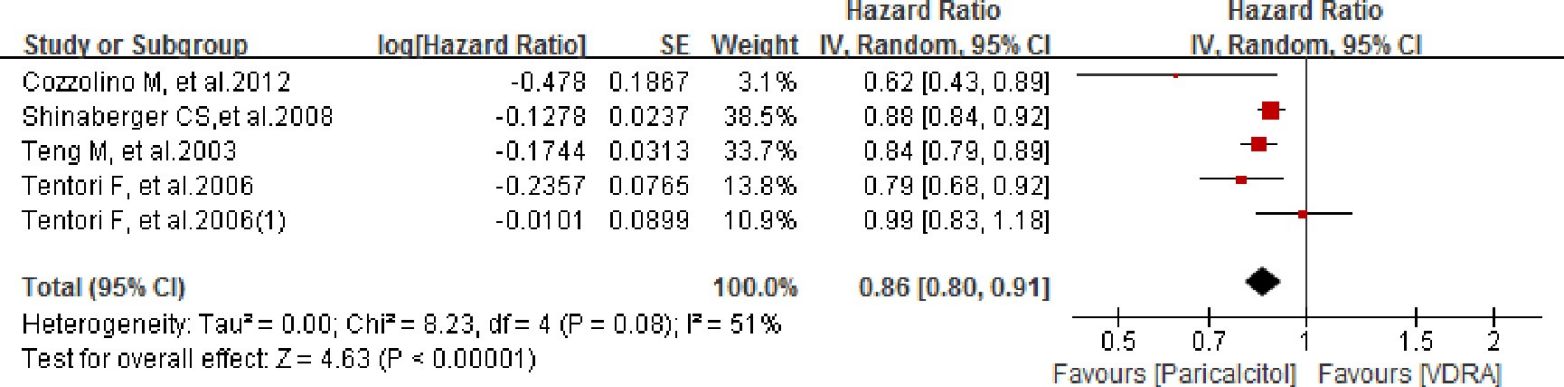
Forest plot of comparison between paricalcitol and VDRA regarding to overall survival.

Four observational studies with five sets of data were included for comparison of overall survival between paricalcitol and other VDRAs. The study of Tentori et al. (2006) offered two independent data groups and thus we pooled-analyzed both the data. As shown in Fig 4, patients receiving paricalcitol showed better overall survival (OS) compared to patients receiving other VDRAs, with a pooled hazard ratio (HR) of 0.86 (95% CI 0.80–0.91; *P* < 0.00001). As significant heterogeneity was observed (*P*_*h*_ *=* 0.08 and *I*^*2*^ *=* 51%), the pooled analysis was conducted using a random effects model.

### Comparison of iPTH between paricalcitol and other VDRAs

**Fig 5:**
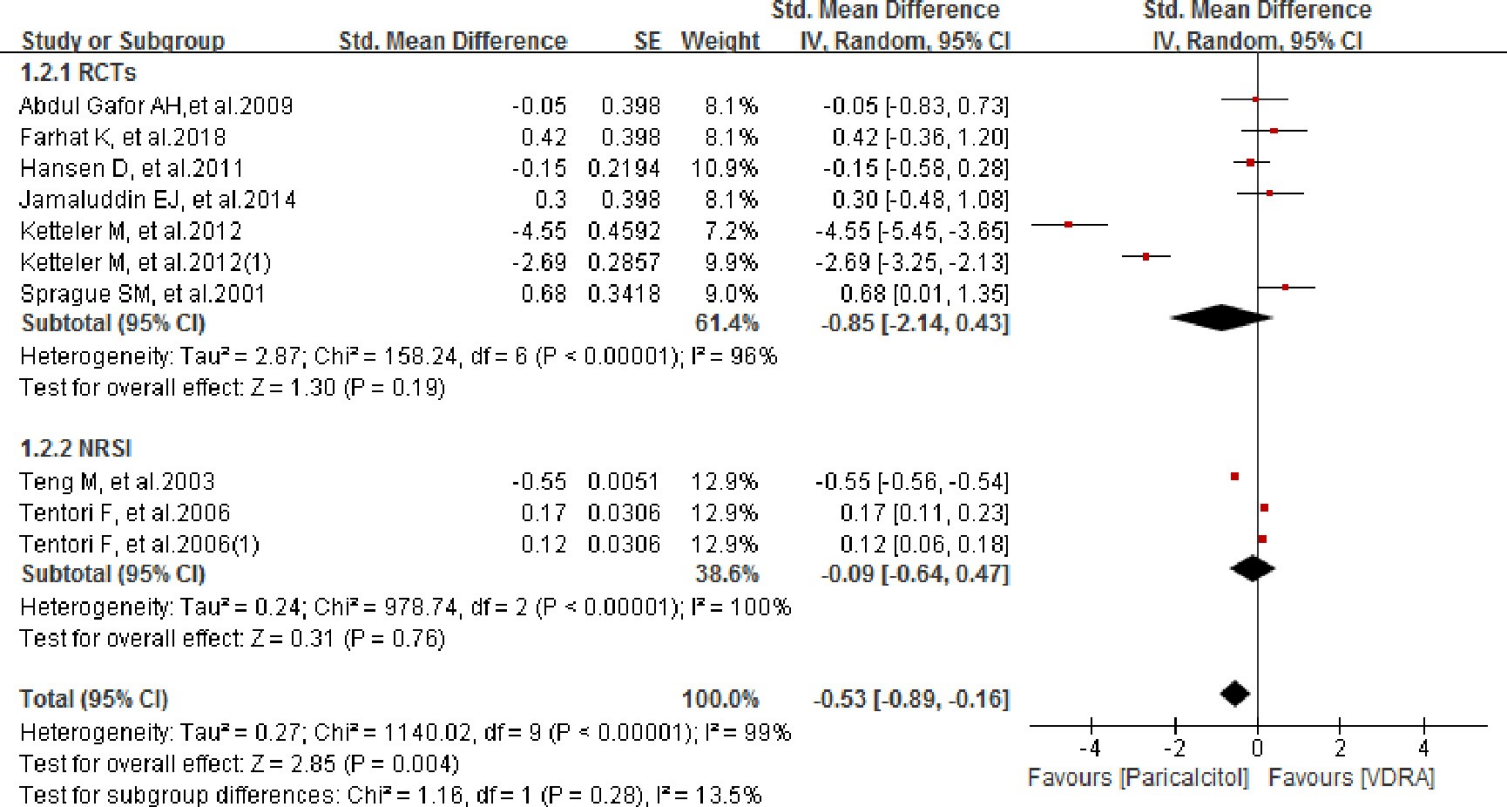
Forest plot of comparison between paricalcitol and VDRA regarding to intact parathyroid hormone level.

We compared the change in iPTH from baseline between paricalcitol and other VDRAs. In the study of Tentori et al. and Ketteler et al., both of them offered two independent data group and thus we pooled-analyzed both the data. As shown in Fig 5, a significant reduction was found in the paricalcitol group compared to the other VDRAs group, with a pooled SMD of -0.53 (95% CI -0.89–-0.16; *P* = 0.004). No significant difference was found in subgroup analyses of RCTs and NRSIs, with pooled SMDs of -0.85 (95% CI -2.14–0.43; *P* = 0.19) and -0.09 (95% CI -0.64–0.47; *P* = 0.76), respectively. For the subgroup analysis of RCTs, we conducted sensitivity analysis by removing each study from the analysis in turn. No single study had a significant effect on the pooled result. However, as we observed in Fig 5, the study of Ketteler M et al.2012 experienced much heterogeneity. When the data of Ketteler M et al.2012 and Ketteler M et al.2012(1) were singly omitted from the analysis list, the heterogeneity was not significantly changed and the pooled results of RCTs group were changed from (SMD -0.85; 95% CI -2.14 to 0.43; *P* = 0.19; *I*^*2*^ = 96%) to (SMD -0.26; 95% CI -1.32 to 0.80; *P* = 0.63; *I*^*2*^ = 94%) and (SMD -0.54; 95% CI -1.80 to 0.72; *P* = 0.40; *I*^*2*^ = 95%) respectively. However, when we removed both Ketteler M et al.2012 and Ketteler M et al.2012(1) meanwhile from the list, the heterogeneity was significantly changed from *I*^*2*^ = 96% to *I*^*2*^ = 22%. The pooled result showed good stability with no significant change observed (SMD 0.18; 95% CI -0.15 to 0.51; *P* = 0.29). We also repeated the analysis after deleting studies with a Jadad score less than 2 [16, 21]. There was no significant difference between groups, with a pooled SMD of -1.24 (95% CI -2.95–0.46; *P* = 0.15).

### Comparison of serum calcium level between paricalcitol and other VDRAs

**Fig 6:**
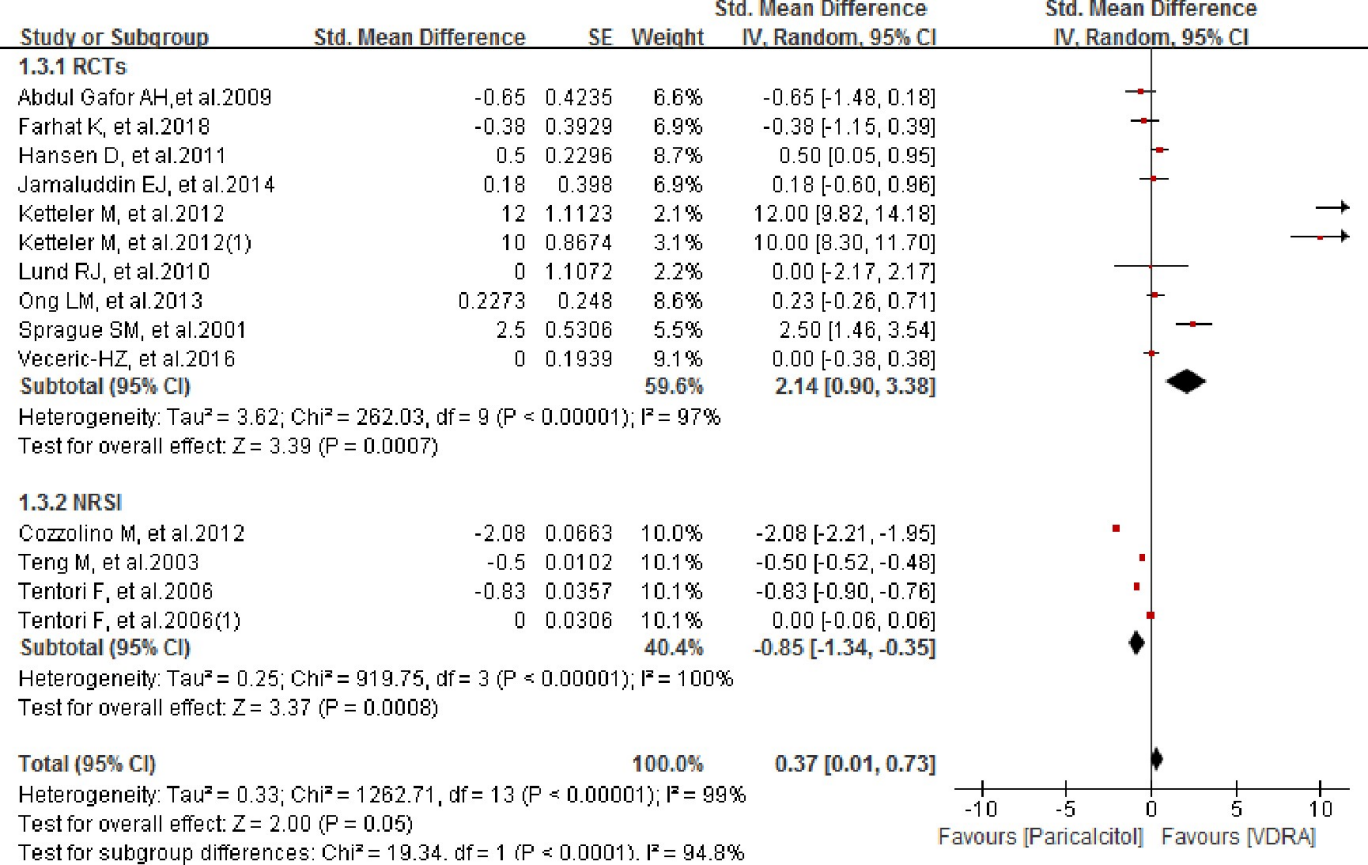
Forest plot of comparison between paricalcitol and VDRA regarding to serum calcium level.

We compared changes in serum calcium level from baseline between paricalcitol and other VDRAs; In the study of Tentori et al. and Ketteler et al., both of them offered two independent data group and thus we pooled-analyzed both the data. No significant difference was found, with a pooled SMD of 0.37 (95% CI 0.01–0.73; *P* = 0.05; Fig 6). A subgroup analysis of RCTs showed that changes in serum calcium levels were significantly larger in the paricalcitol group, with a pooled SMD of 2.14 (95% CI 0.90–3.38; *P* = 0.0007). However, the changes were significantly smaller in the paricalcitol group in a subgroup analysis of NRSIs, with a pooled SMD of -0.85 (95% CI -1.34 to -0.35; *P* = 0.0008). Regarding subgroup analysis of RCTs, sensitivity analysis showed that the results were stable and were not significantly affected by the omission of any single study, except that of Abdul Gafor et al. [16]. Excluding this study changed the pooled SMD from 0.37 (95% CI 0.01–0.73; *P* = 0.05) to 0.44 (95% CI 0.07–0.81; *P* = 0.02). However, when the data of Ketteler M et al.2012 and Ketteler M et al.2012(1) were singly omitted from the analysis list, the pooled results of RCTs group were not significantly changed with the pooled SMDs changing from 2.14 (95% CI 0.90 to 3.38; *P* = 0.0007; *I*^*2*^ = 97%) to 1.19 (95% CI 0.18 to 2.20; *P* = 0.02; *I*^*2*^ = 95%) and 1.21 (95% CI 0.24 to 2.18; *P* = 0.01; *I*^*2*^ = 94%) respectively. However, when we removed both Ketteler M et al.2012 and Ketteler M et al.2012(1) meanwhile from the list, the result was significantly changed to SMD of 0.26 (95% CI -0.21 to 0.74; *P* = 0.28; *I*^*2*^ = 75%). Omitting all studies with a Jadad score < 2 [16, 21, 24, 29] from the overall analysis also had a significant effect; the pooled SMD changed such that there was a significant difference between the groups in change in serum calcium level from baseline (pooled SMD = 0.56 (95% CI 0.12–0.99; *P* = 0.01).

### Comparison of serum phosphorus level between paricalcitol and other VDRAs

**Fig 7:**
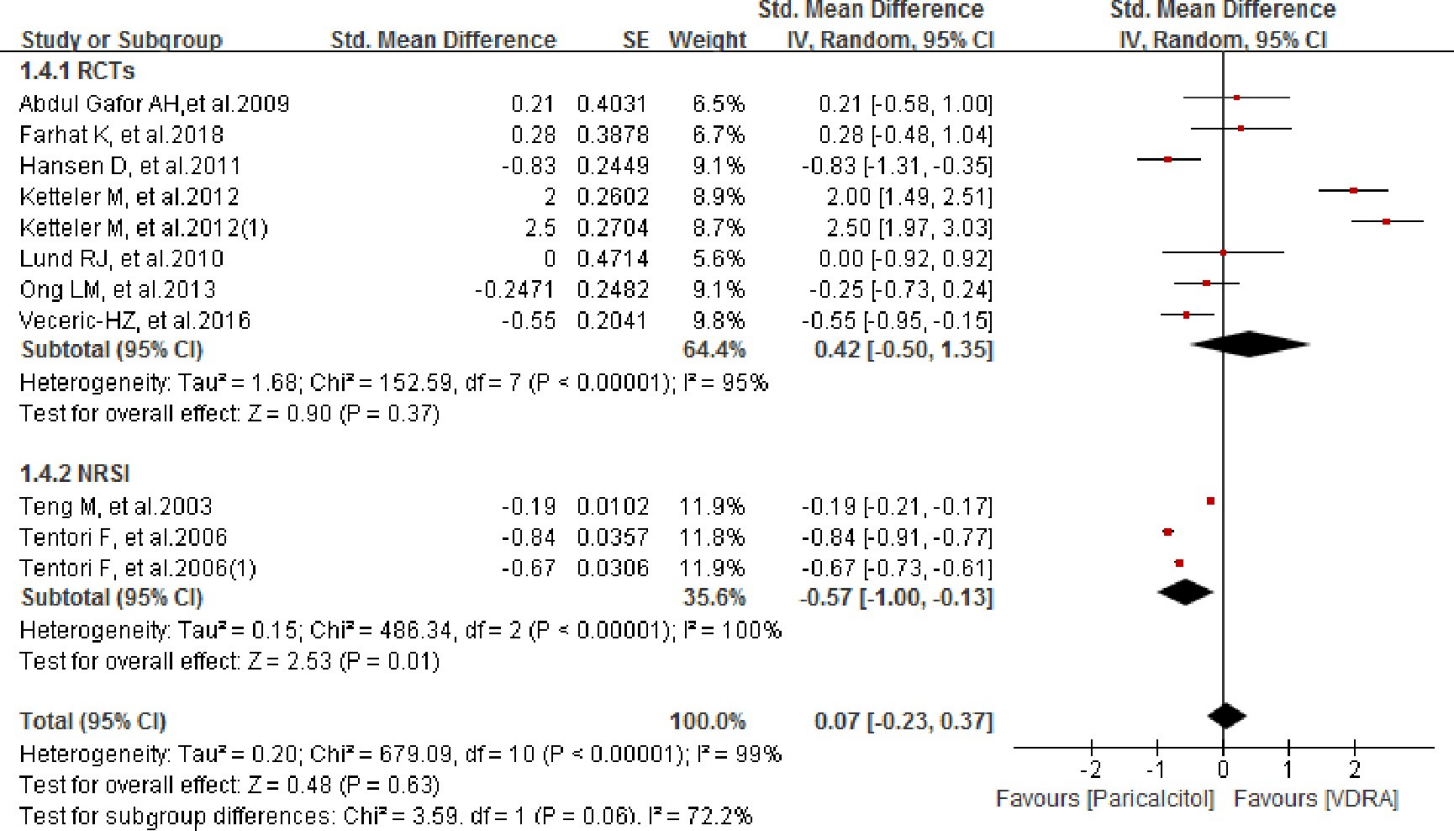
Forest plot of comparison between paricalcitol and VDRA regarding to serum phosphorus level.

We also compared changes in serum phosphorus levels from baseline between the paricalcitol and other VDRAs groups. In the study of Tentori et al. and Ketteler et al., both of them offered two independent data group and thus we pooled-analyzed both the data. As shown in Fig 7, no significant difference between groups was found. The pooled SMD for change in serum phosphorus level from baseline was 0.07 (95% CI -0.23–0.37; *P* = 0.63) in the analysis of all studies, versus 0.42 (95% CI -0.50–1.35; *P* = 0.37) in the analysis of RCTs. The subgroup analysis of NRSIs showed that the change in serum phosphorus level was significantly smaller in the paricalcitol group than in the other VDRAs group, with a pooled SMD of -0.57 (95% CI -1.00– -0.13; *P* = 0.0008). Sensitivity analysis showed that, overall, the results were highly stable, and were not significantly affected by the omission of any single study. However, when the data of Ketteler M et al.2012 and Ketteler M et al.2012(1) were singly omitted from the analysis list, the pooled results of RCTs group were not significantly changed with the pooled SMDs changing from 0.42 (95% CI -0.50 to 1.35; *P* = 0.37; *I*^*2*^ = 95%) to 0.19 (95% CI -0.71 to 1.10; *P* = 0.68; *I*^*2*^ = 94%) and 0.12 (95% CI -0.66 to 0.90; *P* = 0.77; *I*^*2*^ = 92%) respectively. However, when we removed both Ketteler M et al.2012 and Ketteler M et al.2012(1) meanwhile from the list, the heterogeneity was significantly changed from *I*^*2*^ = 95% to *I*^*2*^ = 50%. However, the pooled result still showed good stability with no significant change observed (SMD -0.29; 95% CI -0.63 to 0.05; *P* = 0.10). When studies with a Jadad score < 2 were excluded, the overall and RCT subgroup analyses showed no significant group differences in the change in serum calcium level from baseline. The pooled SMDs for the overall and RCT subgroup analyses were 0.18 (95% CI -0.17–0.53; *P* = 0.30) and 0.80 (95% CI -0.60–2.20; *P* = 0.26), respectively.

### Comparison of other outcomes between paricalcitol and other VDRAs

Finally, we compared seven outcomes for the RCTs between the paricalcitol and other VDRAs groups. As shown in Table 2, there was a significant increase in the Ca × P product for the paricalcitol group compared to the other VDRAs group (SMD 2.13; 95% CI 0.19–4.07; *P* = 0.031). However, no significant difference between groups was observed for iPTH control rate (RR 1.03; 95% CI 0.94–1.13; *P* = 0.52), hypercalcemia (RR 0.93; 95% CI 0.73–1.20; *P* = 0.59), hyperphosphatemia (RR 0.95; 95% CI 0.77–1.18; *P* = 0.65), ≥ 50% reduction in iPTH (RR 0.99; 95% CI 0.76–1.31; *P* = 0.967), adverse events (RR 1.02; 95% CI 0.93–1.12; *P* = 0.674), or rate of elevated Ca × P product (RR 1.08; 95% CI 0.81–1.44; *P* = 0.60).

**Table 2:** The pooled results of other outcomes for the comparison of paricalcitol versus VDRA.

Outcomes	Number of studies	Number of participants	Pooled results		
			Effect estimates	95% CI	*P* value
iPTH controlling rate	7	964	RR 1.03	0.94, 1.13	0.52
Hypercalcemia	5	696	RR 0.93	0.73, 1.20	0.59
Hyperphosphatemia	3	604	RR 0.95	0.77, 1.18	0.65
Change in Ca×P product	5	470	SMD 2.13	0.19, 4.07	0.031*
≥50% reduction in iPTH	3	367	RR 0.99	0.76, 1.31	0.967
Adverse events	3	593	RR 1.02	0.93, 1.12	0.674
Elevated Ca×P product	3	415	RR 1.08	0.81, 1.44	0.60

VDRA, vitamin D receptor activator; iPTH, intact parathyroid hormone; Ca, calcium; P, phosphorus; RR, relative risk; SMD, standard mean difference; CI, confidence intervals.

### Subgroup analysis and publication bias

Subgroup analysis was conducted to compare the paricalcitol and other VDRAs groups in terms of the reduction in iPTH from baseline. As Table 3 shows, there was no significant difference between the paricalcitol group and other VDRAs group in subgroup analyses of route of administration (intravenous, oral), sample size (≤ 100 pts, > 100 pts), baseline iPTH level (≤ 68.4 pmol/L, > 68.4 pmol/L) or Jadad score (≤ 2, > 2).

**Table 3:** Subgroup analysis of the comparison of paricalcitol versus VDRA regarding reduction of iPTH from baseline.

Outcomes	Number of studies	Number of participants	Pooled results		
			RR	95% CI	*P* value
Routine of administration					
Intravenous	3	604	1.07	0.91, 1.26	0.42
Oral	2	92	0.82	0.61, 1.11	0.21
Sample size					
≤ 100 pts	3	178	0.98	0.75, 1.28	0.87
> 100 pts	2	518	1.02	0.79, 1.32	0.88
Baseline of iPTH level					
≤ 68.4 pmol/L	3	407	0.97	0.78, 1.20	0.77
> 68.4 pmol/L	2	289	1.09	0.84, 142	0.51
Jadad score					
≤ 2	2	92	0.82	0.61, 1.11	0.21
> 2	3	604	1.07	0.91, 1.26	0.42

VDRA, vitamin D receptor activator; iPTH, intact parathyroid hormone; RR, relative risk; CI, confidence intervals.

**Fig 8:**
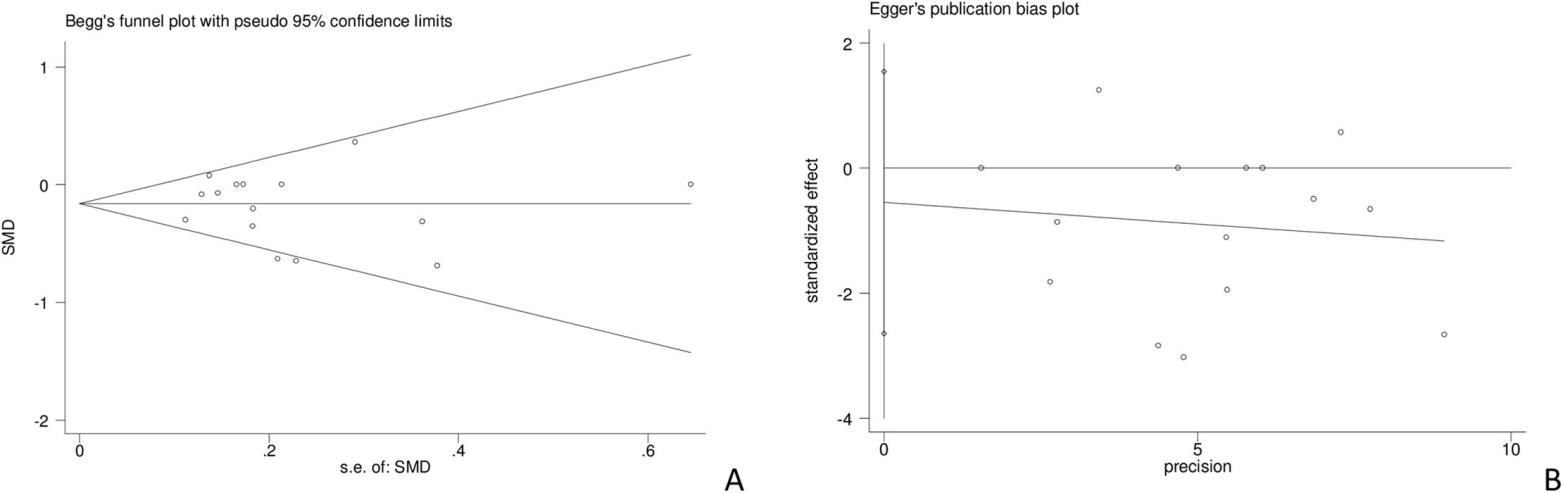
Begg’s funnel plot (A) and Egger’s publication bias plot (B) for detecting publication bias.

Begg’s funnel and Egger’s publication bias plots were generated to assess publication bias in the included studies. No clear evidence of publication bias was observed, for the Egger’s test being 0.97 (95% CI -2.645 to 1.548; *P* = 0.581); as shown in Fig 8A and 8B.

Vitamin D deficiency is not uncommon in the general population, but is seen much more frequently in patients with ESRD, with a prevalence reported to exceed 80% [47]. Low vitamin D levels are associated with hyperparathyroidism, low calcium and calcitriol serum levels, female gender, obesity, and insufficient sunlight exposure [48]. Previous studies have found that vitamin D deficiency in patients on incident hemodialysis is associated with an increased early mortality rate, and that vitamin D supplementation significantly improves cardiac dysfunction and survival in patients undergoing dialysis [49, 50]. Paricalcitol, as a new VDRA, has been reported to be more effective in reducing calcium and phosphorus levels in patients undergoing hemodialysis. However, the efficacy and safety of paricalcitol are controversial. Therefore, we conducted this analysis to compare paricalcitol to other VDRAs in patients undergoing hemodialysis.

The search strategy for this review was comprehensive and systematic, and included hand-searching of the references of included studies and previous systematic reviews. The results of our pooled analysis, which included 15 studies, indicated that paricalcitol was superior to other VDRAs in prolonging OS and reducing iPTH levels in patients undergoing hemodialysis. One present meta-analysis of Liu et al. [51] with thirteen studies involving 112,695 patients showed significant difference between paricalcitol and other VDRAs in overall survival (HR = 0 .86, 95% CI: 0.80, 0.92; *P*<0.001) and the intact para-thyroid hormone (iPTH) (SMD = −0.53, 95% CI: −0.90, −0.17; *P* = 0.004). However, the study made a methodological mistake which pooled-analyzed the data from RCTs and cohort studies. This mistake could lead to serious bias and could influence the pooled results. Thus, we conducted our analysis respectively with RCTs and NRSIs data and found that although a significant difference between groups in reduction in iPTH levels was observed overall, subgroup analyses of RCTs and NRSIs showed no group differences. Regarding the change in serum calcium level from baseline, contradictory results were observed in subgroup analyses of RCTs and NRSI. Post-intervention, the serum calcium level was significantly higher in the paricalcitol group than in the other VDRAs group in the analysis of RCTs, but significantly lower in the analysis of NRSIs. The subgroup analyses included 10 RCTs and 4 retrospective studies. Although RCTs are considered more reliable than retrospective studies, the latter group of studies had a much larger total sample size (*N* = 75,151). This suggests that the analysis of retrospective studies should not be ignored, despite the lower credibility of such studies. The subgroup analysis of NRSIs showed that the change in serum phosphorus level from baseline was significantly smaller in the paricalcitol group than in the other VDRAs group; this was not seen in the RCT subgroup analysis. Liu et al. reported that changes in serum calcium, phosphate, and calcium phosphate levels in patients taking paricalcitol were comparable to those in patients taking other VDRAs [52]. The incidence of adverse events was similar between the groups in that study, consistent with our findings.

In addition, Xie 2017 [51] the compared efficacy of efficacy and safety between paricalcitol and other VDRAs for management of secondary hyperparathyroidism (SHPT) in dialysis patients and found insufficient evidence. Teng et al. conducted a retrospective cohort study comparing the survival rate between paricalcitol (*N* = 29,021) and calcitriol (*N* = 38,783) groups [27]. At the 36-month follow-up, the mortality rates of these two groups were significantly different (RR = 0.80, 95% CI: 0.77, 0.84; *P* = 0.001). In the paricalcitol group, there were 3,417 deaths over 19,031 person-years of observation (0.18 per person-year), compared to 6,805 deaths over 30,471 person-years (0.22 per person-year) in the calcitriol group. Moreover, patients who switched from calcitriol to paricalcitol had a survival benefit exceeding that of those who switched from paricalcitol to calcitriol [27]. In another clinical trial, hemodialysis patients received paricalcitol (*N* = 2,087), doxercalciferol (*N* = 2,432), or calcitriol (*N* = 3,212) [28]. Patients treated with paricalcitol showed a similar mortality rate to that of those treated with doxercalciferol, but a significantly lower mortality rate than that of those receiving calcitriol. At the end of the 37-week follow-up period, the mortality rate (deaths/100 patient-years) was 15.3 (95% CI: 13.6, 16.9) in the paricalcitol group, 15.4 (95% CI: 13.6, 17.1) in the doxercalciferol group, and 19.6 (95% CI: 18.2, 21.1) in the calcitriol group [28]. The HR for paricalcitol versus doxercalciferol was estimated to be 0.99 (95% CI: 0.84, 1.15), which was not statistically significant [28].

In addition, as we know, vitamin D stimulates osteocyte fibroblast growth factor 23 (FGF23) production and may lead to a significant increase of circulating FGF23. Moreover, high FGF23 has been associated to a poor survival in CKD in dialysis patients through its deleterious effects on the cardiovascular system among others. In addition, there are clinical evidence that polymorphism in the FGF23 co-receptor klotho gene might determine the deleterious or beneficial effect of vitamin D on survival. Thus, future studies should consider this factors and conduct further analysis focusing this influence factors.

Several limitations of our analysis should be acknowledged. The main limitation was discrepancies in the measurement methods used among the included studies, which may have biased the analysis. For instance, Abdul Gafor et al. required patients who were on calcitriol (i.v. or p.o.), 1-a calcidol, bisphosphonate, or calcitonin prior to undergo a 2-week washout period to enrolment [16]. In contrast, Farhat et al. prescribed a 6-week washout period and switched patients using high calcium-containing dialysis fluids (1.75 mmol/L) to low calcium fluids (1.25 mmol/L) [19]. A second limitation was differences in the measures among the studies. In Abdul Gafor et al., the i.v. vitamin D dose was increased every 3 weeks to achieve a minimum 50% reduction in serum iPTH [16]. The initial paricalcitol dose was 0.04 ug/kg, which was increased in increments of 0.04 ug/kg every 3 weeks. The initial calcitriol dose was 0.01 ug/kg, which was increased by 0.01 ug/kg every 3 weeks. Doses were reduced if serum iPTH levels decreased to less than 10 pmol/L, or if serum calcium exceeded 2.8 mmol/L (normal range: 2.14–2.58 mmol/L) [16]. The primary efficacy end point was the proportion of subjects in each treatment group who achieved a mean iPTH level of 150–300 pg/mL during the evaluation period (weeks 21–28 of the treatment period) [22]. Secondary analyses were performed to determine the proportions of participants who achieved a ≥ 30 or ≥ 50% reduction from baseline in iPTH, and the proportion who experienced hypocalcemia (mean calcium < 8.4 mg/dL (2.09 mmol/L)) or hypercalcemia (mean calcium level > 10.5 mg/dL (2.61 mmol/L)) during weeks 21–28 [22]. Another limitation of our analysis was that no dichotomous outcomes showed significant differences between groups, despite the significant differences in continuous variables such as iPTH, serum calcium, and phosphorus levels, and the degree of change in Ca × P. The limited number of studies included in the analysis precluded subgroup analysis of dichotomous outcomes. The third important limitation of this meta-analysis is that none of the studies included controlled for circulating levels of 25OHD before treating secondary hyperparathyroidism with active vitamin D analogs. Fourth, we stressed that nine of the 15 studies came from the USA; thus, the results might not hold true for other populations such as Asian or European. Because of the limitation of study number and sample size, we failed to conduct the subgroup analysis according to geographic characteristics. Finally, we did not compare the efficacy of paricalcitol to that other VDRAs after grouping patients by other clinical features that may influence outcomes, such as gender, age, and follow-up time.

## Conclusion

In conclusion, paricalcitol seems superior to other VDRAs in reducing the mortality rate and iPTH levels in patients undergoing hemodialysis. However, the effectiveness of paricalcitol in reducing serum calcium and phosphorus levels needs further exploration. No significant group difference was found in the rate of adverse events. Well-designed prospective studies are needed to compare the effects of different VDRAs, including selective and non-selective VDRAs, in CKD patients undergoing hemodialysis.

## Supporting information

S1 Checklist PRISMA 2009 checklist. (DOC)

S1 Appendix Example electronic search strategies (Medline). (DOCX)
