# Supplementary material for: A comparative analysis of the efficacy and safety of paricalcitol versus other vitamin D receptor activators in patients undergoing hemodialysis: A systematic review and meta-analysis of 15 randomized controlled trials
Source: PLoS One. 2020 May 29;15(5):e0233705. doi: 10.1371/journal.pone.0233705 (PMC7259607; doi:10.1371/journal.pone.0233705)
Supplement: S1 Appendix — (DOCX) [file pone.0233705.s002.docx]

**Example Electronic search strategies (Medline)**

| **Number** | **Search strategy** |
| --- | --- |
| 1 | Dialysis [Mesh] |
| 2 | Ultrafiltration |
| 3 | hemodialysis |
| 4 | haemodialysis |
| 5 | hemodiafiltration |
| 6 | haemodiafiltration |
| 7 | hemofiltration |
| 8 | haemofiltration |
| 9 | vitamin [Mesh] |
| 10 | paricalcitol [Mesh] |
| 11 | paricalcitol* |
| 12 | vitamin D |
| 13 | kidney failure [Mesh] |
| 14 | renal insufficiency |
| 15 | chronic kidney disease |
| **16** | 1 OR 2 OR 3 OR 4 OR 5 OR 6 OR 7 OR 8 |
| **17** | 9 OR 10 OR 11 OR 12 |
| **18** | 13 OR 14 OR 15 |
| **19** | 16 AND 17 AND 18 |
